# Accelerating Synchrotron MX Beamlines: Automated Sample Centering with Machine Learning and Bluesky Orchestration

**DOI:** 10.1063/4.0000839

**Published:** 2025-10-27

**Authors:** David Aragao, Martin Savko, Dominic Oram, Kate Smith, William Shepard, Ralf Flaig

**Affiliations:** 1 Diamond Light Source, Didcot, Oxforshire, United Kingdom; 2 Synchrotron SOLEIL, Gif-sur-Yvette, NA, France; 3 Australian Synchrotron, Australian Nuclear Science and Technology Organisation, Clayton, Victoria, Australia

## Abstract

Recent advances in synchrotron technology have shifted the bottleneck in macromolecular crystallography (MX) data collection from sample irradiation to other time-consuming steps, such as sample alignment. This project, being prototyped by the I04 beamline at the Diamond Light Source^(a)^, is developing a high-throughput automated pipeline for precise and efficient sample centering using machine learning. The pipeline incorporates image analysis and leverages the 'murko' software^(b)^, a machine learning model for sample identification and centering, developed by Proxima 2 at the SOLEIL synchrotron^(c)^. Device management is handled using Ophyd, and the orchestration of plans is managed by Bluesky^(d)^, ensuring a robust and flexible system. By integrating this technology with Kubernetes and Docker containers, we ensure portability and scalability for seamless deployment across various synchrotron facilities. This project is also part of a larger project that aims to significantly accelerate MX workflows, enabling faster and more reliable data collection to support diverse research applications using the bluesky/ophyd technology^(e)^. Future endeavors may involve generating machine learning-based tomographic masks of the sample holder and protein crystal, facilitating optimal X-ray centering grid determination and starting angle selection.
